# Preparation of Quaternary Ammonium Separation Material based on Coupling Agent Chloromethyl Trimethoxysilane (KH-150) and Its Adsorption and Separation Properties in Studies of Th(IV)

**DOI:** 10.3390/molecules29133031

**Published:** 2024-06-26

**Authors:** Zheng Wang, Xique Wu, Meichen Liu, Xiaoqiang Zhao, Haichao Wang, Xiangfu Meng, Xiaofei Zhang

**Affiliations:** 1 Department of Chemical Engineering, Hebei Petroleum University of Technology, Chengde 067000, China; wcyzwz7566395@163.com (Z.W.); 18331295975@163.com (X.W.); zxq830919@163.com (X.Z.); haiwhc1@aliyun.com (H.W.); 2 Department of Radiochemistry, China Institute of Atomic Energy, Beijing 102413, China; liu13069831833@163.com; 3 College of Environmental and BioEngineering, Putian University, Putian 351100, China

**Keywords:** Th(IV), separation material, coupling agent, quaternary ammonium, adsorption

## Abstract

In this research, the authors studied the synthesis of a silicon-based quaternary ammonium material based on the coupling agent chloromethyl trimethoxysilane (KH-150) as well as its adsorption and separation properties for Th(IV). Using FTIR and NMR methods, the silicon-based materials before and after grafting were characterized to determine the spatial structure of functional groups in the silicon-based quaternary ammonium material SG-CTSQ. Based on this, the functional group grafting amount (0.537 mmol·g^−1^) and quaternization rate (83.6%) of the material were accurately calculated using TGA weight loss and XPS. In the adsorption experiment, the four materials with different grafting amounts showed different degrees of variation in their adsorption of Th(IV) with changes in HNO_3_ concentration and NO_3_^−^ concentration but all exhibited a tendency toward anion exchange. The thermodynamic and kinetic experimental results demonstrated that materials with low grafting amounts (SG-CTSQ_1_ and SG-CTSQ_2_) tended to physical adsorption of Th(IV), while the other two tended toward chemical adsorption. The adsorption mechanism experiment further proved that the functional groups achieve the adsorption of Th(IV) through an anion-exchange reaction. Chromatographic column separation experiments showed that SG-CTSQ has a good performance in U-Th separation, with a decontamination factor for uranium in Th(IV) of up to 385.1, and a uranium removal rate that can reach 99.75%.

## 1. Introduction

Extensive research has been conducted in recent years on separating and adsorbing various elements in the end products of nuclear fuel. Thorium, which exists in trace amounts in the end uranium products, is not only an important element that needs to be controlled in the product but also holds significant importance in nuclear fuel reprocessing and environmental management [1]. According to the statistics, nuclear power plants worldwide produce over 10,500 tons of spent fuel annually, including more than 170 tons of thorium-232 that can be further utilized [2]. Due to technological limitations, this spent fuel undergoes simple reprocessing and is then solidified and buried as nuclear waste. This nuclear waste not only poses environmental safety hazards but also leads to significant resource wastage [3]. While the previous literature has documented the recovery and utilization of trace thorium in simulated reprocessing samples, enhancing separation methods to improve the recovery rate remains a challenge in spent fuel reprocessing [4,5].

In previous reprocessing studies, the separation of thorium in the sample was mainly achieved through extraction and ion exchange methods [6]. The extraction separation method has significant advantages in the separation of actinide trace elements (such as thorium, neptunium, and plutonium) due to its high selectivity and separation efficiency [7]. Thanks to this benefit, various extractants (including chelating ones, phosphorus-based ones, and amine-based ones) have been utilized and progressed to some degree in separating trace actinide elements. For example, Yu Yufu used polyurethane foam loaded with PMBP (an amine-based extractant) to separate trace thorium from a large amount of uranium, followed by further purification using TEVA quaternary ammonium resin. The decontamination factor for uranium from thorium in this method reaches 2.7 × 10^6^ [8]. Hassan S. Ghaziaskar successfully separated thorium from zirconium carbide waste residue by liquid–liquid extraction with trioctylamine after acid leaching. Chuanqin Xia designed and synthesized a ditriamide extractant, which has high selectivity for Th(IV) [9,10]. However, the extraction separation method requires meticulous operations such as shaking and phase separation, leading to long operation times and complex processes. Additionally, for the separation of trace elements, extractants must have extremely high distribution ratios, which limits the variety of available extractants and makes them expensive [11]. The ion-exchange method offers the benefits of easy-to-use equipment and the opportunity for reuse. In the late 20th century, American scientists systematically conducted research on anion-exchange technology for the separation and purification of actinide trace elements, such as R.H. Poirier, who successfully achieved the separation and analysis of trace thorium from a large amount of uranium using pyridine anion-exchange resin [12]. The ion-exchange resins utilized in the ion-exchange technique offer benefits such as high exchange capacity and the ability to be reused; however, their organic framework is easily damaged under extreme conditions of spent fuel reprocessing (high acid concentration, strong radiation), which limits their rapid development in the field of radioactivity [13]. Extraction separation and ion-exchange methods each come with their own set of advantages and limitations. Given this, it is essential to develop and create a reprocessing separation material that integrates the benefits of both, thus possessing the efficiency of the extraction method as well as the convenience of the solid-phase separation method. Additionally, the material’s framework should be resistant to damage. In recent years, many researchers have also tried to develop new separation materials or methods for the separation of thorium. For example, Tonghuan Liu prepared selective cellulose materials after Amidoxime-functionalized cellulose and studied its adsorption and separation properties for Th(IV). Tianxiang Jin modified graphene oxide to obtain materials that can capture thorium ions efficiently. Faur et al. synthesized carbamoyl-methylphosphonated hydrosoluble polymers and investigated their selectivity for Gd(III)/Th(IV)/U(VI) separation [14,15,16]. These studies have made progress and breakthroughs in the separation process of thorium to a certain extent but still cannot overcome the shortcomings of the current separation materials in some aspects.

In nuclear fuel reprocessing, a qualified separation material matrix should have good mechanical properties, acid resistance, and radiation resistance. According to recent years’ studies, silicon-based materials have been widely used in the field of radioactivity due to their excellent performance in various aspects. Zhang et al. grafted a novel functionalized silica composite material (SiAcP) on the surface of porous silica gel using bis(2-methacryloyloxyethyl) phosphate (BMAOP) as a monomer and optimized the synthesis conditions. The adsorption separation behavior of the new material for Sc(III) and Zr(IV) was studied through column experiments, and the results showed that the material exhibited good selective adsorption for Zr(IV) [17]. Chen et al. studied the adsorption behavior of polyamine-grafted silica composite material (SAER) for uranium in neutral and weakly acidic solutions, demonstrating the feasibility of the novel silicon-based composite adsorbent to effectively remove uranium from different aqueous matrices [18]. Compared to the organic framework of ion-exchange resins, silica gel has good mechanical properties, acid resistance, and radiation resistance. The hydroxyl groups on the surface of silica gel can be used for chemically grafting functional monomers, greatly enhancing the stability of functional groups and effectively inhibiting the loss of functional materials [19]. Furthermore, the majority of contemporary methods used to produce porous silica gel yield materials with significant pore size and surface area, offering significant advantages in enhancing the performance of adsorption separation materials. Hence, the utilization of silicon-based materials in the realm of radioactivity holds great promise.

Amine extractants are often used to separate and purify actinides in the field of nuclear fuel reprocessing analysis. Among them, the quaternary ammonium salt extractor is widely used in the separation of neptunium, thorium, and plutonium in uranium in the reprocessing process due to its short equilibrium time and high selectivity (Figure S1: the relationship between the partition coefficient K_d_ of various ions on TEVA and the concentration of nitric acid). For example, TEVA (trioctylmethyl ammonium chloride or ammonium nitrate) raffinate resin has a remarkable effect in the analysis and pretreatment of thorium [20,21]. In our previous research, we utilized a coupling agent to chemically graft and modify the surface of porous silica gel (mesoporous) and investigated its grafting mechanism and grafting rate [22]. Based on this theory, this study grafted the coupling agent chloromethyl trimethoxysilane (KH-150) on the surface of porous silica gel, then used trioctylamine to quaternize the modified silica gel to obtain a silica-based quaternized separation material for the study of Th(IV) adsorption and separation. The research content is divided into three parts. Firstly, the materials before and after quaternization were characterized by FTIR, NMR, XPS, and BET to determine the connection mode and spatial structure of functional groups on the silica gel surface, and then the grafting amounts of functional groups under different synthesis conditions were determined through TGA, thereby obtaining the theoretical adsorption capacity of the materials. Secondly, through the study of Th(IV) adsorption behavior, the adsorption reaction mechanism of the silica-based quaternized separation material for Th(IV) was determined, additionally, thermodynamics and kinetics of adsorption were explored. Finally, the material was used for the separation study of trace Th(IV) in simulated reprocessing of uranium samples. This study offers fresh insights and perspectives for enhancing and optimizing thorium separation materials in reprocessing activities, along with introducing a novel approach for utilizing silica gel-based materials in the realm of reprocessing separation.

**Figure 1:**
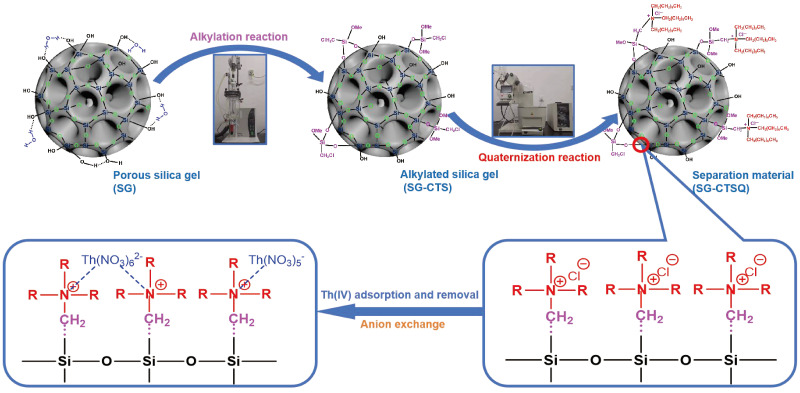
Preparation procedure of SG-CTSQ and the adsorption process of Th(IV).

Figure 1 illustrates the preparation process of SG-CTSQ and the adsorption process of Th(IV).

## 2. Results and Discussion

### 2.1. Characterization of Silicon-Based Quaternized Material

**Figure 2:**
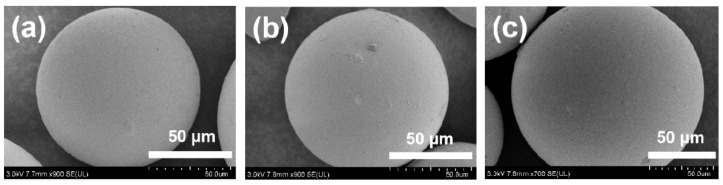
SEM images of SG (**a**), SG-CTS (**b**), and SG-CTSQ (**c**).

The SEM images of SG (a), SG-CTS (b), and SG-CTSQ (c) are shown in Figure 2. It can be seen from SEM images that the surface of porous silica gel particles has no significant changes in either the alkylation process or quaternization process. This is because both processes are small-molecule grafting processes, which is not enough to cause significant surface changes. However, with the progress of grafting, the appearance color of the material changed significantly, that is, the alkylated material showed light green and the quaternized material showed yellow (Figure S3), which are due to external manifestations caused by changes in the molecular structure of the surface of the material [23]. In order to further prove that the surface structure of porous silica gel changes, the hydrophilic properties of the material were investigated. As can be seen from the figure (Figure S3), SG particles all sink to the bottom in deionized water; however, SG-CTS and SG-CTSQ particles are mostly suspended or float in water, which proves that a large number of organic hydrophobic groups already exist on the surface of the material, providing preliminary proof that the coupling agent and trioctylamine have been successfully grafted to the surface of the silica gel.

**Figure 3:**
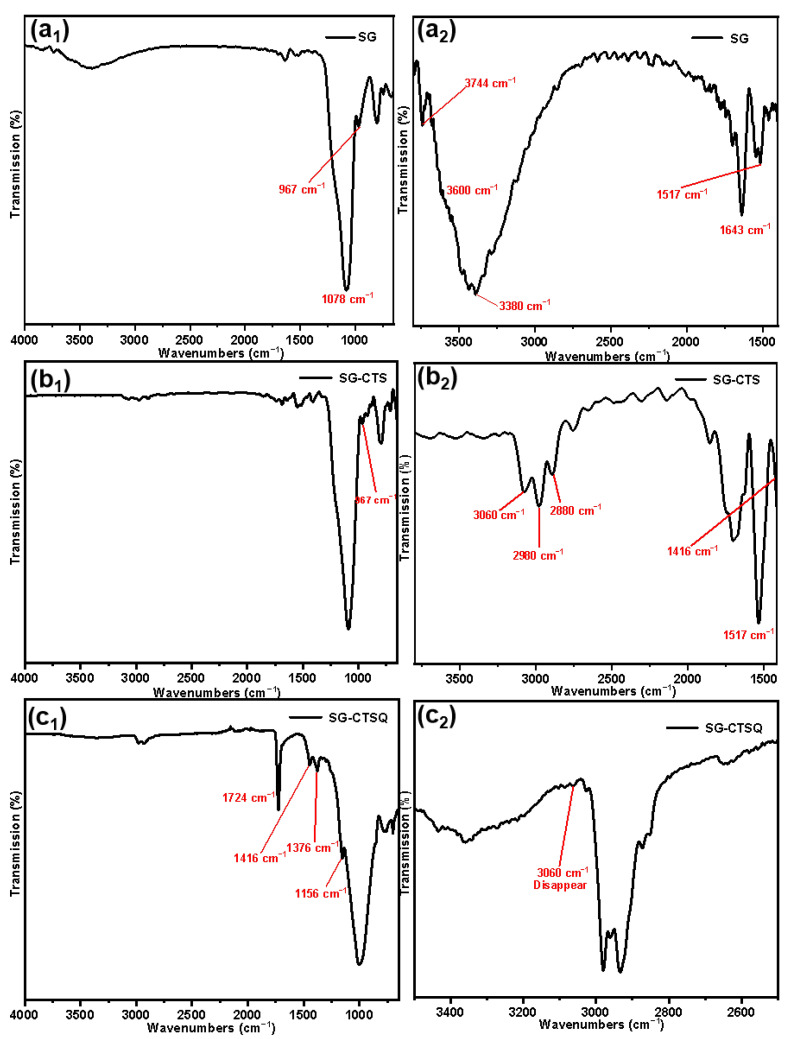
FTIR spectra of the SG (**a**), SG-CTS (**b**), and SG-CTSQ (**c**).

The FTIR spectroscopies of SG (a), SG-CTS (b), and SG-CTSQ (c) are depicted in Figure 3. For SG, in Figure 3a_1_, 967 cm^−1^ is the flexural vibration peak of −OH, and 1078 cm^−1^ is the stretching vibration peak of Si−O−Si [24]. In Figure 3a_2_ (enlarged portion of 1400–3800 cm^−1^ in Figure 3a_1_), 1643 cm^−1^ is the flexural vibration peak of H_2_O and 1517 cm^−1^ is the in-plane deformation vibration peak of −OH. The broad peak around 3380 cm^−1^ is the stretching vibration peak of the double-base −OH and H_2_O, and 3744 cm^−1^ is the stretching vibration peak of the free-type −OH [25]. In addition, there is a peak at about 3600cm^−1^ which is the stretching vibration peak of hydrogen-bonded −OH, which is obscured by the large peak of 3380cm^−1^ but is faintly visible [26]. Figure 3a shows that there are three different types of −OH on the surface of SG, namely free-type −OH, hydrogen-bonded −OH, and double-base −OH; in addition, there is a certain amount of bound water on the surface of SG.

For SG-CTS, in Figure 3b_1_, due to the large amount of −OH involved in the reaction, the peaks of 967 cm^−1^ and 3744 cm^−1^ were significantly weakened. In Figure 3b_2_ (enlarged portion of 1400–3800 cm^−1^ in Figure 3b_1_), 3060 cm^−1^ is the stretching vibration peak of C−H in −CH_2_Cl, 2980 cm^−1^ and 2880 cm^−1^ are the stretching vibration peaks of C−H in −CH_3_, and 1416 cm^-1^ is the in-plane bending vibration peak of C-H in −CH_3_ [27]. In addition, the bending vibration peak of water at 1643 cm^−1^ and the stretching vibration peak of water at around 3380 cm^−1^ disappear, which indicates that there are a large number of organic hydrophobic groups on the surface of silica gel and the binding water is significantly reduced [28]. The above analysis shows that the characteristic peaks of the groups in the coupling agent KH-150 are all present in the SG-CTS spectrum, which indicates that the coupling agent KH-150 has been successfully grafted on the surface of the silica gel.

For SG-CTSQ, in Figure 3c_1_, due to the introduction of a large amount of octyl (containing −CH_3_ and −CH_2_−) on the surface of silica gel after the quaternization reaction, the in-plane bending vibration peak of C−H in −CH_3_ was more obvious at 1416 cm^−1^ and the out-of-plane bending vibration peak of C−H in −CH_3_ appeared at 1156 cm^−1^, which was previously covered by the large peak of 1078 cm^−1^ [29]. The in-plane bending vibration peak at 1376 cm^−1^ is of C−H in −CH_2_−. In addition, the sharp peak of 1724 cm^−1^ is the C−N stretching vibration peak corresponding to quaternary ammonium nitrogen. In Figure 3c_2_ (enlarged portion of 2500–3500 cm^−1^ in Figure 3c_1_), the most obvious change is the disappearance of the C−H stretching vibration peak (3060 cm^−1^) in −CH_2_Cl, which indicates that this group has participated in the quaternization reaction [30]. The above analysis shows that trioctylamine was successfully present on the surface of the silica gel and quaternized with coupling agent molecules.

**Figure 4:**
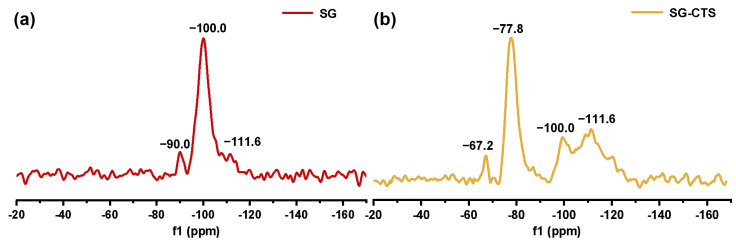
^29^Si-MAS NMR spectra of SG (**a**) and SG-CTS (**b**).

The ^29^Si-MAS NMR spectra of SG and SG-CTS are shown in Figure 4. For SG, in Figure 4a, −90.0 ppm is the absorption peak of Si corresponding to the double-base −OH, −100.0 ppm is the absorption peak of Si corresponding to free-type −OH and hydrogen-bonded −OH, and −111.6 ppm is absorption peak of Si corresponding to siloxane groups [31]. After the grafting coupling agent reaction, for SG-CTS, in Figure 4b, the absorption peak at −90.0 ppm disappeared, indicating that double-base −OH almost completely participated in the reaction. The intensity of the absorption peak at −100.0 ppm decreased significantly, indicating that most of the independent −OH participated in the grafting reaction [32]; however, lots of −OH participated in the reaction, increasing the number of Si corresponding to siloxane groups Si−(OSi)_4_, which enhanced the intensity of the absorption peak at −111.6 ppm. In addition, −67.2 ppm and −77.8 ppm are peaks of Si of the coupling agent KH-150 with two different connection modes, respectively [33]. Among them, −67.2 ppm corresponds to the coupling agent molecule with only one chemical bond connected to the silica gel, and −77.8 ppm corresponds to the coupling agent molecule with two chemical bonds connected to the silica gel [34]. Based on the above analysis, the possible surface structures of SG and SG-CTS are shown in Figure 6a and 6b, respectively.

**Figure 5:**
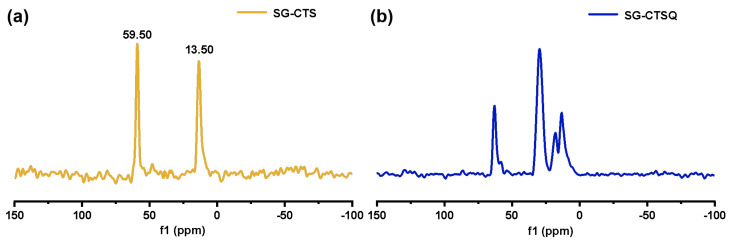
^13^C-NMR spectra of SG-CTS (**a**) and SG-CTSQ (**b**).

**Figure 6:**
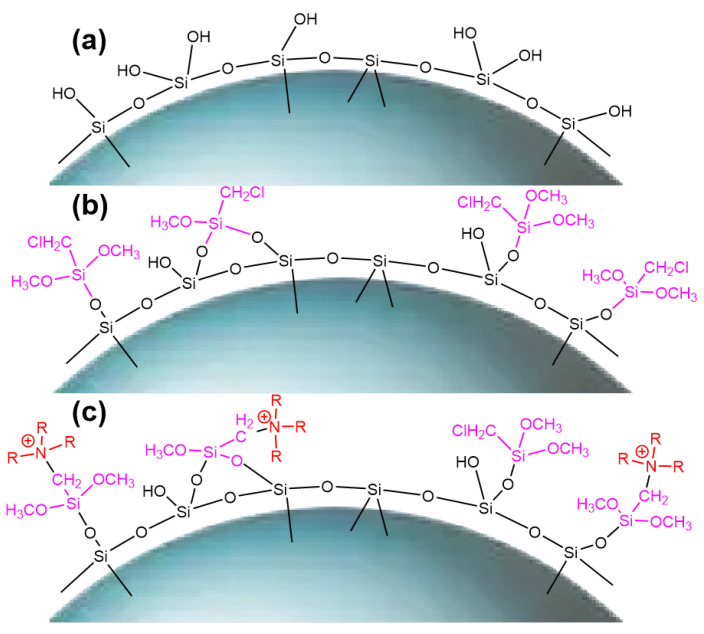
Surface morphology of SG (**a**), SG-CTS (**b**), and SG-CTSQ (**c**).

The ^13^C-NMR spectra of SG-CTS and SG-CTSQ are shown in Figure 5. For SG-CTS, in Figure 5a, 13.5 ppm is the absorption peak of C corresponding to the −OCH_3_, and 59.5 ppm is the absorption peak of C corresponding to the −CH_2_Cl [35]. For SG-CTSQ, in Figure 5b, the peak at 59.5 ppm disappeared, which indicates that a large number of −CH_2_Cl groups disappeared [36]. Nevertheless, the peak observed at 62.5 ppm corresponds to the C absorption peak associated with quaternary ammonium nitrogen, suggesting that trioctylamine has effectively undergone quaternization with the −CH_2_Cl group in the coupling agent molecule. In addition, 18.2 ppm is the characteristic peak of C corresponding to −CH_3_ in trioctylamine, and 29.3 ppm is the characteristic peak of C corresponding to −CH_2_− in trioctylamine [37]. Based on the above analysis, the possible surface structure of SG-CTSQ is shown in Figure 6c.

**Figure 7:**
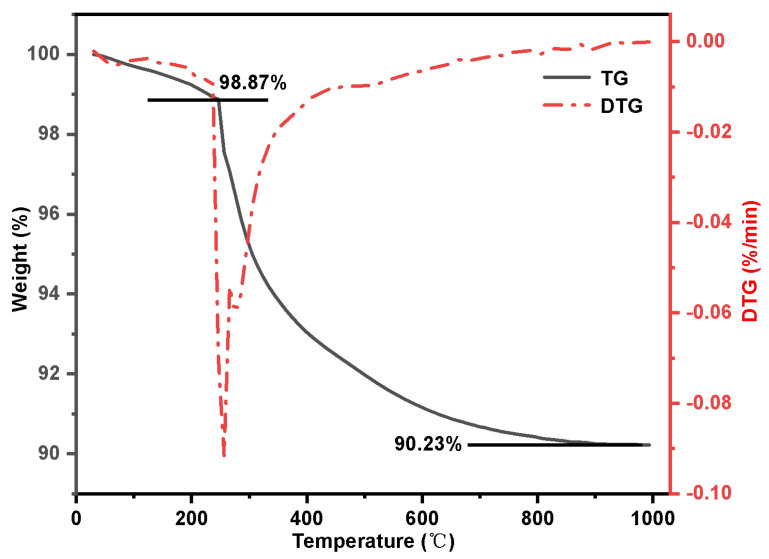
Thermogravimetric curve of SG-CTS.

Figure 7 displays the TG-DTG results for alkylated silica gel SG-CTS. The image clearly shows that the weight reduction occurring between 30 and 246 °C is due to the dissociation of the organic solvent xylene from the surface of silica gel. The weight loss in the range of 246–905 °C is the decomposition process of −OCH_3_ and −CH_2_Cl on the coupling agent molecules. In this thermogravimetric process, the weight of organic group decomposition is m_1_=8.64%. By substituting it into Equation (5), the grafting amount G(mmol·g^−1^) of quaternary ammonium groups can be calculated as follows:

(1) $$G=\frac{m_{2}-0.0864}{0.9136M}*10^{3}$$

**Figure 8:**
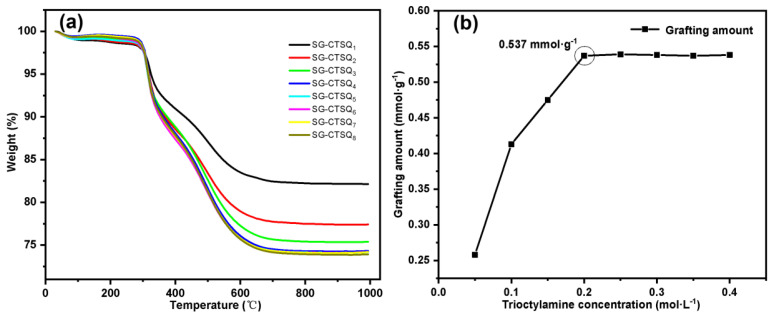
Thermogravimetric curves (**a**) and grafting amount trend (**b**) of SG-CTSQ_(1–8)_.

The TG-DTG results of materials synthesized under different concentrations of trioctylamine (SG-CTSQ_1_ to SG-CTSQ_8_) are shown in Figure 8a. When the concentration of trioctylamine is low, the thermal weight loss increases rapidly with the increase in the concentration. When the concentration of trioctylamine participating in the reaction reaches 0.2 mol·L^−1^, the thermal weight loss reaches the maximum. Thereafter, with the increase in trioctylamine concentration, the trend of the thermogravimetric curve remained the same. Since the grafting amount G(mmol·g^−1^) of quaternary ammonium groups depend on the thermogravimetric weight loss m_2_, the change trend of grafting amount can be obtained by Equation (1), as shown in Figure 8b. When the trioctylamine concentration was 0.05 mol·L^−1^, 0.1 mol·L^−1^ and 0.15 mol·L^−1^, the grafting amounts were 0.258 mmol·g^−1^, 0.413 mmol·g^−1^, and 0.475 mmol·g^−1^, respectively. When the trioctylamine concentration was greater than 0.2 mol·L^−1^, the surface reaction site reached saturation, and the grafting amount reached the maximum value (0.537 mmol·g^−1^) and maintained equilibrium.

**Figure 9:**
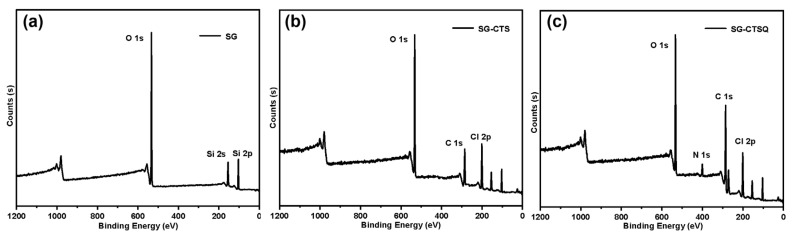
XPS spectra of SG (**a**), SG-CTS (**b**), and SG-CTSQ (**c**).

In order to explore the changes in elements and functional groups on the surface of the materials, and to obtain the actual maximum quaternization grafting rate, XPS tests were performed on SG (a), SG-CTS (b), and SG-CTSQ_4_ (c) (the material with the highest grafting amount mentioned above), as shown in Figure 9. For SG, in Figure 9a, 104 eV and 155 eV are the 2p and 2s absorption peaks of Si, respectively; 533 eV and 980 eV are 1s and 0 KLL absorption peaks of O, respectively [38]. As can be seen from the figure, SG contains only two elements, Si and O. The XPS spectrogram of SG-CTS were changed after the reaction with the coupling agent KH-150 (chloromethyl trimethoxy-silane). In Figure 9b, 201 eV is the 2p peak of Cl, and 286 eV is the 1s peak of C, which indicates that the coupling agent molecule was successfully present on the silica gel surface [39]. After quaternization (Figure 9c), the most obvious change was that the C 1s peak at 286 eV was significantly enhanced, and the 1s peak of quaternary ammonium nitrogen appeared at 401 eV [40].

**Figure 10:**
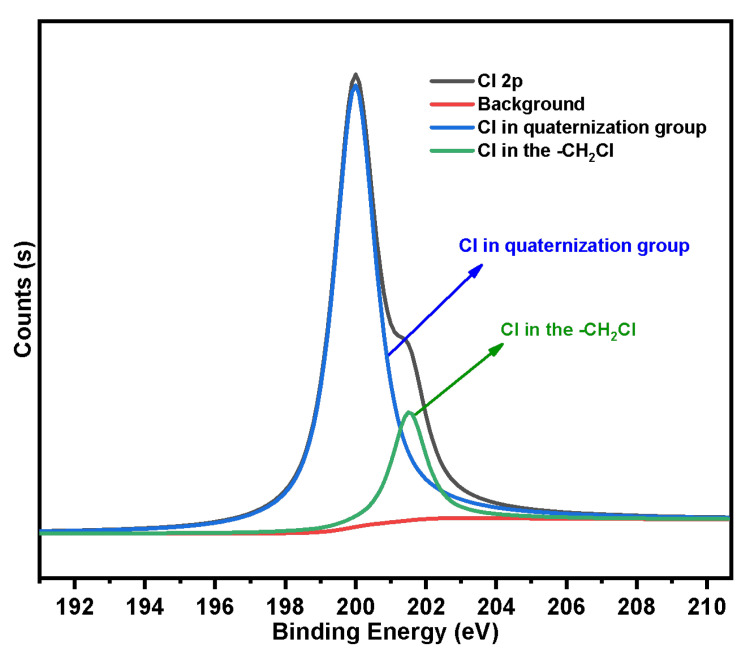
The deconvolution of SG-CTSQ Cl 2p spectra.

In order to further determine the actual maximum grafting rate of the quaternization reaction (namely, the utilization of coupling agent on SG-CTS), we fitted the Cl 2p absorption peak of SG-CTSQ. After quaternization, the Cl in the material may be linked in the following two ways: -CH_2_Cl not involved in the reaction or Cl in the quaternization group. Two deconvoluted peaks centered at 201.5 eV and 199.9 eV were assigned to the above two connection methods, respectively [41]. The Cl 2p spectra of SG-CTSQ are illustrated in Figure 10. According to the fitting results, the peak area ratios of the two groups are 16.4% and 83.6%, respectively. The maximum grafting rate of quaternization was 83.6%.

Combined with the previous TG-DTG results, it can be seen that under this maximum grafting rate (83.6%), the corresponding grafting amount (the number of quaternizing groups per unit mass of the material) is 0.537 mmol·g^−1^. However, the adsorption effect is not only related to the number of functional groups but is also affected by other factors, which will be discussed later in the paper.

**Figure 11:**
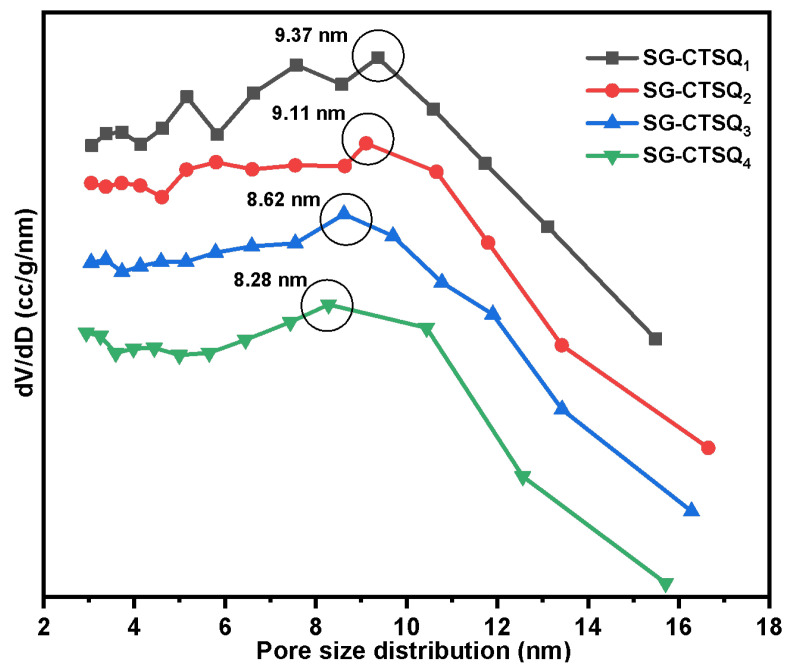
Pore size distribution of SG-CTSQ_1_ to SG-CTSQ_4_.

Figure 11 displays the pore size distribution of SG-CTSQ with varying degrees of grafting; the nitrogen sorption isotherm was put in Figure S4. The results of calculating the maximum probability apertures and specific surface areas of the four materials based on the BET method and BJH method are shown in Table 1. As the grafting amount increases, the material’s specific surface area and pore size decrease gradually. It is obvious that the existence of quaternary ammonium groups occupies a certain surface space of the material. However, even the material with the highest grafting amount (SG-CTSQ_4_) has a pore size greater than 8 nm and a specific surface area greater than 400 m^2^·g^−1^. The above results show that the material still has a large pore size and specific surface area after quaternization, which can be used for adsorption experiments and studies.

### 2.2. Study on the Adsorption and Separation Properties of Th(IV) by Silicon-Based Quaternary Ammonium Material (SG-CTSQ)

The effect and mechanism of the adsorbed material depend on a number of factors, such as the number of functional groups, pore size, specific surface area, steric hindrance, etc., which determines whether the adsorption process is physical adsorption, chemical adsorption, or mixed adsorption type. Hence, this section examined four materials (SG-CTSQ_1_ to SG-CTSQ_4_) with varying degrees of quaternization grafting. The investigation encompassed acidity tests, adsorption thermodynamics analysis, adsorption kinetics experiments, study of adsorption mechanisms, and separation experiments.

**Figure 12:**
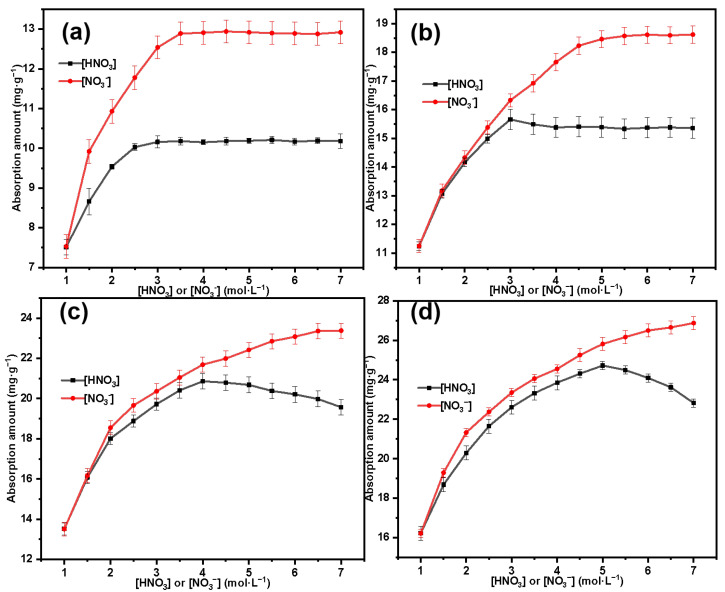
Effects of HNO_3_ and NO_3_^−^ concentrations on adsorption of Th(IV) by SG-CTSQ_1_ (**a**), SG-CTSQ_2_ (**b**), SG-CTSQ_3_ (**c**), and SG-CTSQ_4_ (**d**).

Effects of HNO_3_ and NO_3_^−^ concentrations on adsorption of Th(IV) by SG-CTSQ:

The variation in the adsorption amount of Th(IV) by the material is shown in Figure 12, where the black line represents the change in adsorption amount with the concentration of HNO_3_, and the red line represents the change in adsorption amount with the concentration of NO_3_^−^. For the material SG-CTSQ_4_ with the highest grafting amount (Figure 12d), when the HNO_3_ concentration is in a lower range (1–5 mol·L^−1^), the adsorption amount of Th(IV) by SG-CTSQ_4_ gradually increases with increasing acid concentration; however, as the concentration continues to increase, the adsorption amount shows a decreasing trend, which may be due to the increase in H^+^ concentration or NO_3_^−^ concentration. To analyze this reason, we set the H^+^ concentration at 1 mol·L^−1^ and investigated the change in adsorption amount with NO_3_^−^ concentration under this condition (red line). As the NO_3_^−^ concentration increases, there is no inflection point in the change in Th(IV) adsorption amount by SG-CTSQ_4_, indicating that the adsorption process is influenced by the variation in H^+^ concentration. According to the complexation behavior of Th(IV), it can form complex anions [Th(NO_3_)_6_]^2−^ and [Th(NO_3_)_5_]^−^ with NO_3_^−^, thus, being adsorbed through anion exchange [42]. In this process, the higher the NO_3_^−^ concentration, the greater the proportion of complex anions, making it easier to be adsorbed; however, when the acidity of the system increases, H^+^ also participates in complexation to a certain extent, forming particles such as H_2_Th(NO_3_)_6_ that cannot participate in anion-exchange reactions [10]. Based on the above analysis, we preliminarily believe that the adsorption of Th(IV) by SG-CTSQ_4_ tends to the form of anion exchange.

For SG-CTSQ_1_-SG-CTSQ_3_ (Figure 12a–c), the adsorption curves show significant changes due to the decrease in grafting amount (reduction in quaternary ammonium groups). Firstly, the inflection point of the black line (concentration change curve of HNO_3_) gradually shifts to the left as the material grafting amount decreases, which indicates that fewer functional groups (quaternary ammonium groups) can reach the chemical adsorption saturation state quickly. In other words, a smaller number of reaction sites are more easily able to reach chemical equilibrium. This means that as the H^+^ concentration increases, even if there is only a small change in the concentration of complex anions of Th(IV), it will still have a significant impact on the adsorption effectiveness of the material. Secondly, the change at the inflection point becomes gradually less noticeable, especially for SG-CTSQ_1_ (Figure 12a) since with the increase in HNO_3_ concentration, there is no longer a decreasing trend in adsorption amount. This is because, with the reduction in chemical adsorption sites, the physical adsorption effect becomes more pronounced (due to the characteristics of a porous matrix and large specific surface area), which to some extent masks the chemical adsorption effect.

**Figure 13:**
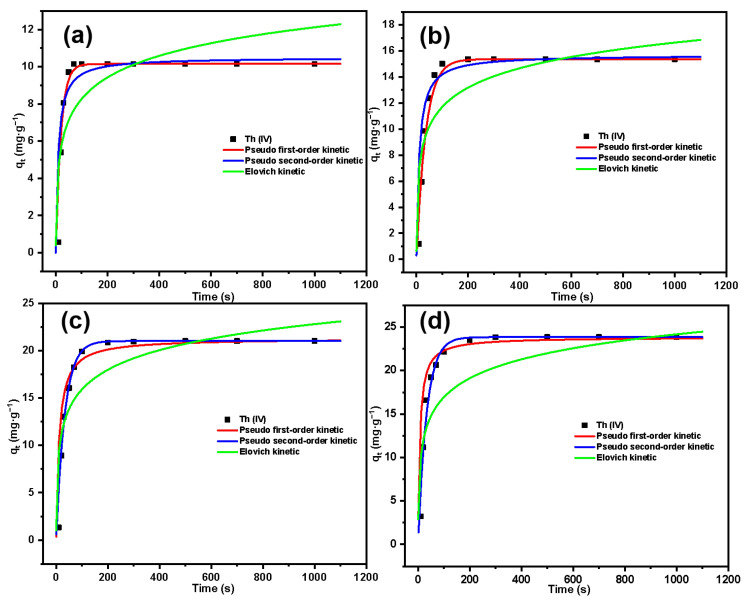
Adsorption kinetics fitting results of Th(IV) on SG-CTSQ_1_ (**a**), SG-CTSQ_2_ (**b**), SG-CTSQ_3_ (**c**), and SG-CTSQ_4_ (**d**).

Study on the kinetics of Th(IV) adsorption by SG-CTSQ:

The variation in Th(IV) adsorption by SG-CTSQ with time is shown in Figure 13. The adsorption kinetics result was analyzed by the pseudo-first-order kinetic model (the reaction rate is linearly related to the concentration of a reactant, and this model is based on the fact that the rate-determining step is a physical process); the pseudo-second-order kinetic model (the reaction rate is linearly related to the concentration of two reactants, and this model is based on the fact that the rate-determining step is a chemical reaction); and the Elovich kinetic model (the Elovich model is suitable for processes with irregular data or with large activation energy) [43,44]. The kinetic parameters for the adsorption of Th(IV) are listed in Table 2.

From the parameters in the table, it can be observed that for the material SG-CTSQ_1_ with the lowest grafting rate, its adsorption kinetics tend to better fit the pseudo-first-order kinetic model (R^2^ = 0.995) compared to the results of fitting the pseudo-second-order kinetic model (R^2^ = 0.964). This indicates that the rate-limiting step of this adsorption process is a physical process. This is due to the lower content of quaternary ammonium functional groups in SG-CTSQ_1_, where physical adsorption is more pronounced compared to chemical adsorption. However, as the grafting amount increases, the fitting results of the pseudo-first-order model gradually improve, and the pseudo-second-order model gradually decrease. The fitting results of the pseudo-second-order model (R^2^ = 0.981, 0.993) for SG-CTSQ_3_ and SG-CTSQ_4_ show a significant improvement compared to the pseudo-first-order model (R^2^ = 0.957, 0.924), suggesting stronger chemical adsorption during the Th(IV) adsorption process. Furthermore, according to Figure 13a, SG-CTSQ_1_ takes around 60 seconds to achieve equilibrium in Th(IV)adsorption, whereas the other three materials require more than 100 seconds (Figure 13b–d). This also indicates that with an increase in grafting amount, the materials tend to exhibit a greater inclination toward chemical adsorption of Th(IV).

For the Elovich kinetic model, the R^2^ for all four materials are relatively small after fitting, indicating that the adsorption process does not involve a significant activation energy. As the grafting amount increases, the R^2^ of this model gradually rises (0.797, 0.837, 0.882, 0.906), indicating that more activation energy is involved in the process and, thus, suggesting a greater inclination toward chemical adsorption (physical adsorption does not require activation energy) [45].

**Figure 14:**
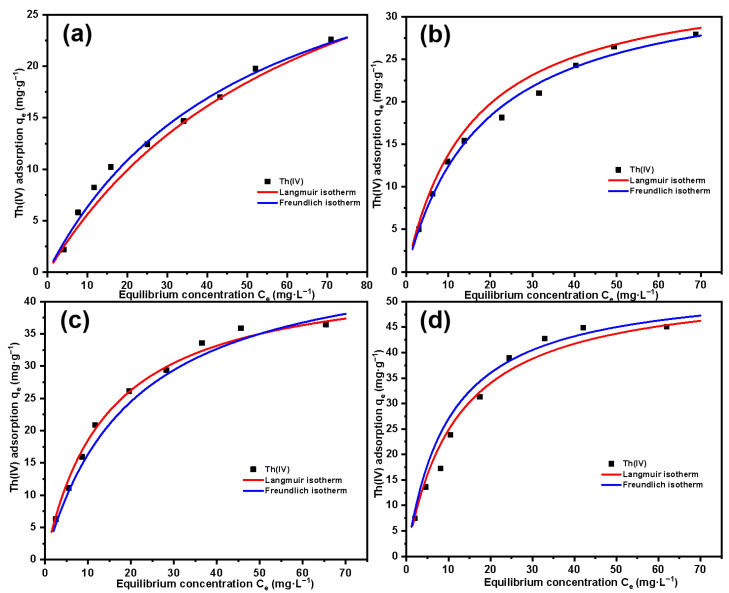
Adsorption isotherm fitting results of Th(IV) on SG-CTSQ_1_ (**a**), SG-CTSQ_2_ (**b**), SG-CTSQ_3_ (**c**), and SG-CTSQ_4_ (**d**).

Study on the thermodynamic of Th(IV) adsorption by SG-CTSQ:

Figure 14 shows the relationship between the equilibrium adsorption amount of the material for different concentrations of Th(IV) and the equilibrium concentration. The four figures, respectively, represent four kinds of materials with different grafting amounts. It can be clearly seen that for each material, with the increase in Th(IV) concentration, the adsorption amount increases and gradually tends to equilibrium, which indicates that the adsorption amount of the material for Th(IV) will eventually reach a saturated adsorption amount. With the increase in grafting amount of SG-CTSQ, the saturated adsorption amount of Th(IV) increased significantly, from about 25 mg·g^-1^ to about 45 mg·g^-1^. The Langmuir model and Freundlich model are based on the assumptions of monolayer adsorption and multilayer adsorption, respectively [46,47]. In general, chemical adsorption and physical adsorption are typical monolayer adsorption and multilayer adsorption processes, respectively [48]. Therefore, we can preliminarily determine the type of Th(IV) adsorption on SG-CTSQ based on the fitting results of these two models. The parameters and fitted plots of Langmuir and Freundlich adsorption isotherm models are listed in Table 3 and Figure 14. The fitting result of the Freundlich model (R^2^ = 0.989) for SG-CTSQ_1_ (Figure 14a) indicates superior performance compared to the Langmuir model (R^2^ = 0.957). This suggests that the adsorption of Th(IV) on SG-CTSQ_1_ leans toward multilayer adsorption, implying a preference for physical adsorption by Th(IV) on SG-CTSQ_1_. As the grafting amount increases (as the number of quaternized groups in the material increases), the determination coefficient R^2^ in the Langmuir model fitting gradually increases. At the maximum grafting level (SG-CTSQ_4_, Figure 14d), the Langmuir model’s fitting result (R^2^ = 0.992) significantly outperforms that of the Freundlich model (R^2^ = 0.976). This suggests that as the quaternized groups in the material increase, there is a preference for monolayer adsorption of Th(IV), indicating that chemical adsorption is the predominant mechanism.

**Figure 15:**
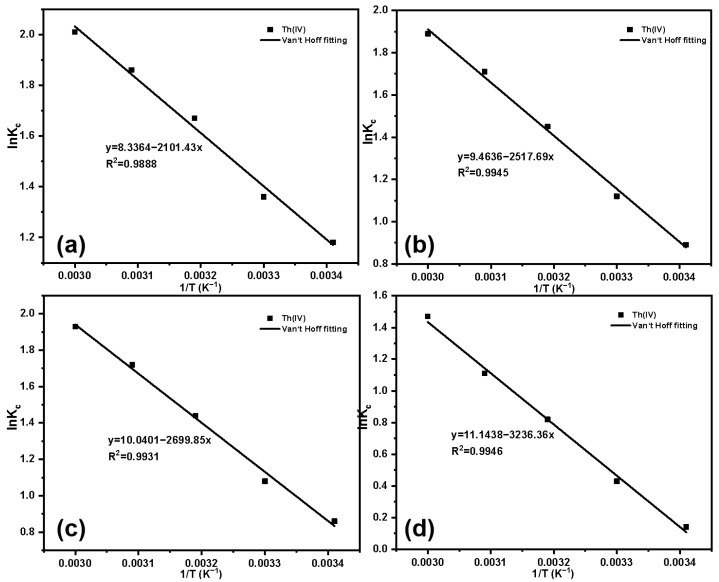
Fitting results of K_c_ and 1/T of Th(IV): SG-CTSQ_1_ (**a**), SG-CTSQ_2_ (**b**), SG-CTSQ_3_ (**c**), and SG-CTSQ_4_ (**d**).

Van’t Hoff’s fitting results and parameters for temperature changes are shown in Figure 15 and Table 4. Based on the parameters in the table, for the four materials with different grafting amounts, ΔG is less than 0 across all temperature ranges, indicating that the adsorption process is spontaneous. Based on Ma et al’s research, the enthalpy change range for physical adsorption falls within 2.10 to 20.90 kJ·mol^−1^. The enthalpy change range for chemical adsorption falls within 20.90 to 418.40 kJ·mol^−1^ [49]. The ΔH for the adsorption of Th(IV) by SG-CTSQ_1_ is 17.47 kJ·mol^−1^ (less than 20.90 kJ·mol^−1^), namely, its adsorption process belongs to physical adsorption. However, for the other three materials, the ΔH is greater than 20.90 kJ·mol^−1^, indicating that their processes are closer to chemical adsorption.

The above analysis shows that in the adsorption process of Th(IV) by SG-CTSQ_1_-SG-CTSQ_4_, the thermodynamic fitting results and kinetic fitting results of adsorptive properties are basically consistent.

Study on adsorption mechanism of Th(IV) on SG-CTSQ:

In this experiment, to study the effect of acidity on adsorption, we initially believed that the functional groups in SG-CTSQ tend to adsorb Th(IV) in the form of anion exchange. To delve deeper into the adsorption mechanism, we carried out the following experiment using SG-CTSQ_4_.

Assuming that the adsorption of Th(IV) by SG-CTSQ has the following reaction formula:

(2) $$(n-4)SiNR_{4}^{+}+Th{\left(NO_{3}\right)}_{n}^{4-n}\overset{K}{\to }(n-4)SiNR_{4}\cdot Th{\left(NO_{3}\right)}_{n}$$

(3) $$K=\frac{\left[(n-4)SiNR_{4}\cdot Th{\left(NO_{3}\right)}_{n}\right]}{{\left[SiNR_{4}^{+}\right]}^{n-4}\cdot [Th{\left(NO_{3}\right)}_{n}^{4-n}]}=\frac{K_{d}}{{\left[SiNR_{4}^{+}\right]}^{n-4}}$$

(4) $$lgK_{d}=lgK+(n-4)lg[SiNR_{4}^{+}]$$

where K is the adsorption equilibrium constant; K_d_ is the distribution ratio; n is the number of complex acid radical ions; [SiNR_4_^+^] is the content of functional groups (mmol); Take lgK_d_ as the ordinate and lg[SiPyR_4_^+^] as the abscissa to draw a straight line, and the value of n can be obtained after fitting.

**Figure 16:**
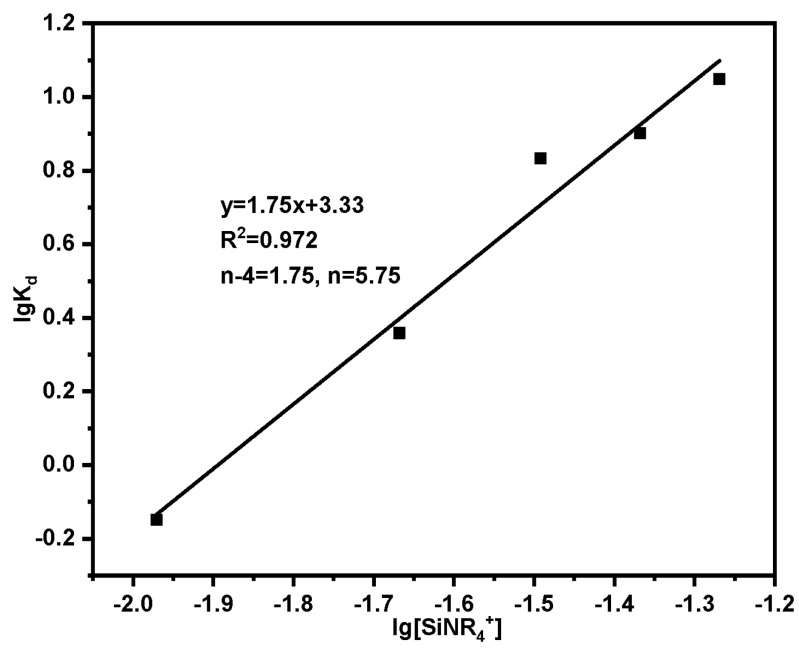
Fitting results of adsorption mechanism.

The results of adsorption using varying dosages of adsorbent are presented in Table 5, with the corresponding fitting outcomes displayed in Figure 16. Based on the slope of the straight line, which is equivalent to n = 5.75, it can be concluded that the adsorption of Th(IV) by SG-CTSQ predominantly involves the complex anions Th(NO_3_)_6_^2−^ and Th(NO_3_)_5_^−^.

Study on separation of uranium and thorium:

Suitable adsorption conditions can improve the adsorption capacity of Th(IV) on SG-CTSQ, thereby improving the recovery rate of Th(IV). According to previous studies, in HNO_3_ system, UO_2_^2+^ mainly exists in the form of UO_2_(NO_3_)^+^ after complexation with NO_3_^−^, and the form of UO_2_(NO_3_)_3_^−^ is less common, so the U(VI) partition coefficient on the material is very low [50]. Based on the static adsorption experiment findings, Th(IV) readily creates complex anions in an HNO_3_ environment, allowing for its absorption onto the material in the chromatography column and, thus, facilitating the separation of uranium and thorium.

**Figure 17:**
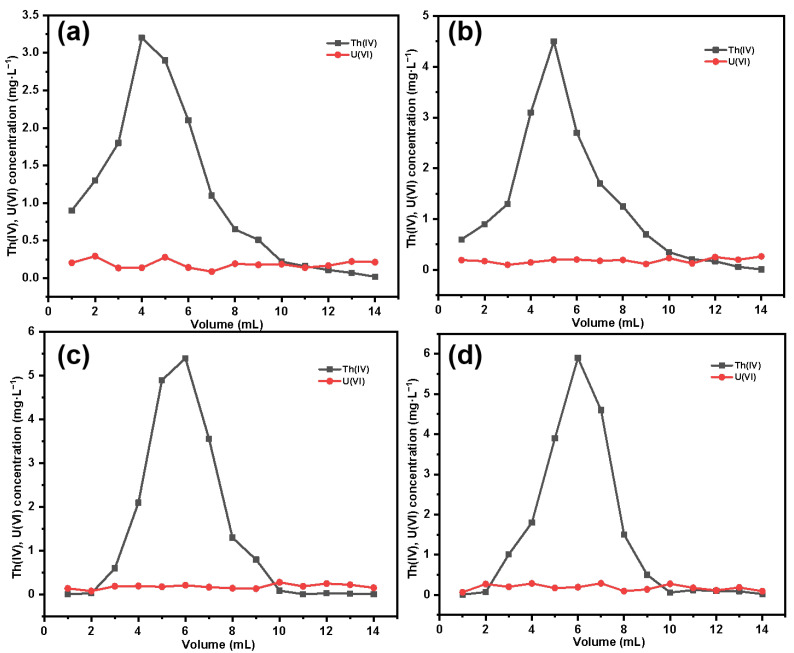
Elution curves for thorium: SG-CTSQ_1_ (**a**), SG-CTSQ_2_ (**b**), SG-CTSQ_3_ (**c**), and SG-CTSQ_4_ (**d**).

After eluting thorium with the corresponding eluent, we obtained the elution curve of Th(IV), as shown in Figure 17. It can be observed that with the increase in grafting amount, there is a noticeable change in the elution curve of thorium. For SG-CTSQ_1_ with a lower grafting amount (Figure 17a), the thorium concentration in the first milliliter of eluate is relatively high, indicating that part of the Th(IV) in the sample is physically adsorbed in the column, which may be because the material has a smaller adsorption distribution coefficient due to a lower content of functional groups, and there is a large amount of free Th(IV) (no ion-exchange reaction has occurred); however, as the material grafting amount increases, this phenomenon gradually disappears (Figure 17b–d). Based on the thorium and uranium concentrations in the original sample and the eluate, the recovery rate of Th(IV) and the decontamination factor of uranium in thorium are shown in Table 6. In terms of Th(IV) decontamination, the newly synthesized silicon-based quaternized material exhibits better decontamination performance compared to the commonly used TEVA resin. Under the same experimental conditions, except for SG-CTSQ_1_—which has a slightly lower decontamination factor than TEVA resin—the other three materials have higher decontamination factors than TEVA resin [51].

## 3. Material and Methods

### 3.1. Materials

Porous spherical silica gel (with a pore size of about 10nm and particle size of about 200 mesh) (Gaokexin, Beijing, China); Xylene (analytical pure), chloromethyl trimethoxysilane (KH-150) (97%), acetonitrile (analytical pure), trioctylamine (98%), anhydrous ethanol, and nitric acid (guarantee reagent) (West Asia Reagent, Chengdu, China); the Th(IV) standard solution and U(VI) standard solution (Department of Radiochemistry, China Institute of Atomic Energy, Beijing, China).

### 3.2. Preparation of Silicon-Based Quaternized Separation Material

The alkylation process of porous silica gel:

Using the coupling agent chloromethyl trimethoxysilane to modify the surface of porous spherical silica gel, alkylated silica gel is obtained. The reaction process is as follows: Soak the porous spherical silica gel in 1 mol·L^−1^ HNO_3_ for 6 h to activate and increase the hydroxyl groups on the surface. The activation process is carried out in an ultrasonic cleaning machine (JP-031S, Skymen, Shenzhen, China) with the temperature set at 60 °C. After rinsing with deionized water, the silica gel is placed in an oven and dried for 24 h to obtain activated silica gel (SG). The activated silica gel (SG) is placed in the air to freely absorb moisture. During this period, continuous weighing is performed until the hydration degree of the silica gel reaches 10% (weight increase of 10%). The activated silica gel (SG) is mixed with xylene solvent and placed in a reaction reactor (DDL-2000, EYELA, Tokyo, Japan) for 15 min of stirring. Then, KH-150 coupling agent is added, and the mixture is stirred at a constant temperature for 24 h (reaction temperature set at 80 °C; KH-150 coupling agent dosage: 0.27 mol·L^−1^). Finally, alkylated silica gel (SG-CTS) is obtained.

The above reaction conditions are the optimal conditions for the alkylation reaction (grafting coupling agent) of silica gel, which was validated in the Authors’ previous research [22].

The quaternization process of silica gel:

The alkylated silica gel (SG-CTS) and acetonitrile solvent are placed in a DDL-2000 reaction reactor and stirred for 15 min at 80 °C to ensure uniform mixing. Then, trioctylamine is added to the reactor and stirred at 80 °C for 24 h at a constant temperature to fully quaternize it. Following the reaction, the material undergoes three washes with ethanol, is dried at 60 °C under vacuum for 24 h, and yields the desired silicon-based quaternized material (SG-CTSQ).

The trioctylamine (0.05 mol·L^−1^, 0.1 mol·L^−1^, 0.15 mol·L^−1^, 0.2 mol·L^−1^, 0.25 mol·L^−1^, 0.35 mol·L^−1^, and 0.4 mol·L^−1^) was employed to conduct the quaternization reaction, leading to the production of quaternized materials exhibiting varying levels of grafting, denoted as SG-CTSQ_1_ to SG-CTSQ_8_, respectively.

### 3.3. Characterization of Silicon-Based Quaternized Material

The characterization of SG-CTSQ includes SEM, FTIR, NMR, XPS, TG-DTG, and BET. The instrument type and parameter Settings used are included in Supplementary Material S1.

### 3.4. Calculation Methods of Grafting Amount and Adsorption Amount

The degree of quaternization on the surface of the silica gel is expressed using the grafting amount G (mmol·g^−1^), which represents the quantity of quaternized groups per gram of SG-CTSQ. Assuming that the 100% weight of SG-CTS is subjected to thermogravimetric analysis, the weight loss is m_1_, and the 100% weight of SG-CTSQ is subjected to thermogravimetric analysis, the weight loss is m_2_. No groups are eliminated during the quaternization reaction so the weight gain of the material in the quaternization process corresponds to the weight of the trioctylamine used in the reaction. After simple derivation, the grafting amount G(mmol·g^−1^) can be calculated as follows:

(5) $$G=\frac{m_{2}-m_{1}}{M(1-m_{1})}*10^{3}$$

where M is the molar mass of trioctylamine.

The total concentrations of Th(IV) were determined by an X-ray fluorescence analyzer (EDX-8100, Shimadzu, Japan). The adsorption amount of Th(IV) in aqueous solution by the SG-CTSQ is calculated by the following Equation:

(6) $$Q=\frac{\left(C_{0}-C_{e}\right)*V}{m}$$

where Q (mg·g^−1^) is the adsorption amount; C_0_ (mg·L^−1^) is the initial concentration of Th(IV) before adsorption; C_e_ (mg·L^−1^) is the concentration of Th(IV) after adsorption; the volume of the solution is denoted as V (L); and the mass of the SG-CTSQ is represented as m (g).

### 3.5. Adsorption Study on Silicon-Based Quaternized Material with Th(IV)

Experiment on the influence of concentration gradients of HNO_3_ and NO_3_^−^:

Th(IV) (20 mg·L^−1^) solution is configured for the HNO_3_ and NO_3_^−^ concentration gradient experiment. Set HNO_3_ or NO_3_^−^ concentration: 1 mol·L^−1^, 1.5 mol·L^−1^, 2 mol·L^−1^, 2.5 mol·L^−1^, 3 mol·L^−1^, 3.5 mol·L^−1^, 4 mol·L^−1^, 4.5 mol·L^−1^, 5 mol·L^−1^, 5.5 mol·L^−1^, 6 mol·L^−1^, 6.5 mol·L^−1^, and 7 mol·L^−1^. Other conditions: temperature, 30 °C; adsorption time, 30 min. After standing for adsorption, filter, measure the equilibrium concentration of Th(IV) with the X-ray fluorescence analyzer, and record it as C_e_. Equation (6) is used to calculate the adsorption amount.

Adsorption kinetic experiments:

The Th(IV) (20 mg·L^−1^) solution is configured for the adsorption kinetics experiment. Set adsorption times: 10 s, 20 s, 30 s, 50 s, 70 s, 100 s, 200 s, 300 s, 500 s, 700 s, and 1000 s. Other conditions: temperature, 30 °C; acidity 4 mol·L^−1^ HNO_3_. At each set time, take a small amount of supernatant and filter it, measure the concentration of Th(IV) with the X-ray fluorescence analyzer, and record it as C_t_. The rate of the adsorption process is determined using the pseudo-first-order, pseudo-second-order, and Elovich kinetic models.

Pseudo-first-order, pseudo-second-order, and Elovich kinetic models are given as Equations (7)–(9) [43,44]:

(7) $$q_{t}=q_{e}\left(1-exp\left(-k_{1}t\right)\right)$$

(8) $$q_{t}=\frac{q_{e}^{2}k_{2}t}{1+q_{e}k_{2}t}$$

(9) $$q_{t}=(1+\beta_{E})ln(1+\alpha_{E}\beta_{E}t)$$

where q_t_ (mg·g^−1^) is the adsorption amount at time t (s); q_e_ (mg·g^−1^) is the equilibrium adsorption amount; k_1_ (s^−1^) denotes the pseudo-first-order kinetic rate constant; k_2_ (g·s·mg^−1^) represents the pseudo-second-order kinetic rate constant; β_E_ (g·mg^−1^) represents the desorption constant associated with the activation energy of chemisorption; α_E_ (mg·(g·s)^−1^) signifies the initial adsorption rate.

Adsorption thermodynamic experiments:

The Th(IV) solutions used for adsorption isotherm studies are as follows: 5 mg·L^−1^, 10 mg·L^−1^, 15 mg·L^−1^, 20 mg·L^−1^, 30 mg·L^−1^, 40 mg·L^−1^, 50 mg·L^−1^, 60 mg·L^−1^, and 80 mg·L^−1^. Other conditions: temperature, 30 °C; acidity 4 mol·L^−1^ HNO_3_; adsorption time, 30 min. After standing for adsorption, filter, measure the equilibrium concentration of Th(IV) with the X-ray fluorescence analyzer, and record it as C_e_. Equation (6) is used to calculate the adsorption amount.

The Langmuir and Freundlich adsorption isotherm models are given as Equations (10) and (11) [46,47]:

(10) $$q_{e}=\frac{q_{max}bC_{e}}{1+bC_{e}}$$

(11) $$q_{e}=K_{f}C_{e}^{n_{f}}$$

where q_e_ (mg·g^−1^) is the equilibrium adsorption amount; q_max_ (mg·g^−1^) is the maximum adsorption amount; C_e_ (mg·g^−1^) is the equilibrium concentration; b is the constant; K_f_ is the Freundlich constant; and n_f_ is the concentration index.

The Th(IV) (20 mg·L^−1^) solution is configured for the adsorption heat experiment. Set temperatures: 20 °C, 30 °C, 40 °C, 50 °C, and 60 °C. Other conditions: acidity 4 mol·L^−1^ HNO_3_; adsorption time, 30 min. After standing for adsorption, filter, measure the equilibrium concentration of Th(IV) with the X-ray fluorescence analyzer, and record it as C_e_. Equation (6) is used to calculate the adsorption amount. The Van’t Hoff equation was used to fit the adsorption amount at different temperatures to obtain the thermodynamic parameters ∆H and ∆S. The change in Gibbs free energy ∆G at various temperatures was determined using the Gibbs equation. Van’t Hoff and Gibbs equations are given as Equations (12) and (13) [52,53]:

(12) $$lnK_{C}=-(\frac{\Delta H}{R})\frac{1}{T}+\frac{\Delta S}{R}$$

(13) ΔG=ΔH−TΔS

(14) $$K_{C}=\frac{C_{s}}{C_{e}}$$

where C_s_ is the concentration of the solid surface at the adsorption equilibrium; and C_e_ is the concentration in the solution at the adsorption equilibrium.

Study on the adsorption mechanism of Th(IV) by SG-CTSQ:

The Th(IV) (20 mg·L^−1^) solution was configured to explore the adsorption mechanism of Th(IV) on SG-CTSQ. Determine the adsorbent dosages: 20 mg, 40 mg, 60 mg, 80 mg, and 100 mg. Other conditions: temperature, 30 °C; acidity 4 mol·L^−1^ HNO_3_; adsorption time, 30 min. After standing for adsorption, filter, measure the equilibrium concentration of Th(IV) with the X-ray fluorescence analyzer, and record it as C_e_. Equation (6) is used to calculate the adsorption amount.

Separation experiment of trace thorium in the uranium matrix:

A certain amount of SG-CTSQ was loaded into an 8 mL extraction chromatography column and passed through the column with 4 mol·L^−1^ HNO_3_ to make it pre-equilibrated for use. The typical flow rate of the chromatographic column is approximately 0.5 mL per minute. Prepare the U-Th mixed samples with the following concentrations U at 1000 mg·L^−1^, Th at 20 mg·L^−1^, and HNO_3_ at 4 mol·L^−1^.

Transfer 1 mL of the mentioned U-Th sample through a chromatographic column. A total of 4 mol·L^−1^ HNO_3_ was used to elute the uranium; then, 0.2 mol·L^−1^ HNO_3_-0.2 mol·L^−1^ Na_2_C_2_O_4_ was used to elute the thorium and the eluent of thorium was collected, which totaled 14 mL. The X-ray fluorescence analyzer was used to measure the concentrations of uranium and thorium, and the elution curves for both elements were generated. The formula for calculating the decontamination factor of uranium in thorium is as follows:

(15) $$DF=\frac{U\;content\;in\;the\;sample/Th\;content\;in\;the\;sample}{U\;content\;in\;eluent/Th\;content\;in\;eluent}$$

Establishment of the Th standard curve:

Thorium standard solutions were prepared by diluting a 500 mg·L^−1^ Th(IV) standard solution to achieve mass concentrations of 5 mg·L^−1^, 10 mg·L^−1^, 20 mg·L^−1^, 40 mg·L^−1^, 60 mg·L^−1^, and 80 mg·L^−1^. The fluorescence intensity A_Th_ (au) of the standard series solution was determined by X-ray fluorescence spectroscopy, and the standard curve was drawn as A_Th_ versus C_Th_ (Th concentration).

The standard curve equation for Th is in Figure S2.

## 4. Conclusions

This study investigated the synthesis of silicon-based quaternized materials based on the coupling agent chloromethyl trimethoxysilane (KH-150) and their adsorption and separation performance for Th(IV). In this synthesis process, materials SG-CTSQ_1_ to SG-CTSQ_4_ with different grafting amounts were synthesized by controlling the concentration of trioctylamine. The NMR characterization was used to determine the molecular structure of the organic groups attached to the surface of porous silica gel, the results show that the coupling agent molecules exist in two ways on the surface of silica gel: it is connected with silica gel through a single chemical bond; it is connected with silica gel by two chemical bonds. Trioctylamine quaternary ammoniated with -CH_2_Cl on the silica gel surface. According to the results of XPS and TGA, the maximum quaternization rate of the coupling agent can reach 83.6%, and the grafting amount of quaternary ammonium groups at 0.537 mmol·g^−1^. In the acidity experiment, the four materials with different grafting amounts showed different degrees of variation in their adsorption of Th(IV) with changes in HNO_3_ concentration and NO_3_^−^ concentration; all exhibited a tendency toward anion exchange. In addition, the adsorption results are also affected by physical factors (pore size, specific surface area, etc.) to a certain extent. The thermodynamic and kinetic experimental results demonstrated that materials with low grafting amounts (SG-CTSQ_1_ and SG-CTSQ_2_) tended to physical adsorption of Th(IV), while the other two tended toward chemical adsorption; with the increase in the grafting amount of SG-CTSQ, the saturated adsorption amount of Th(IV) increased significantly, from about 25mg·g^-1^ to about 45mg·g^-1^. The adsorption mechanism experiment further proved that the functional groups achieve the adsorption of Th(IV) through an anion-exchange reaction. Chromatographic column separation experiments showed that SG-CTSQ has a good performance in U-Th separation, with a decontamination factor for uranium in Th(IV) of up to 385.1, and the uranium removal rate can reach 99.75%. This study provides new ideas and perspectives for the development and optimization of thorium separation materials in reprocessing work, meanwhile offering a detailed approach to the surface modification and application of silica-based materials.

## Figures and Tables

**Table 1:** Specific surface area and pore size with the changes in grafting amount.

Species	Grafting Amount (mmol·g^−1^)	Pore Size (nm)	Specific Surface Area (m^2^·g^−1^)
SG-CTSQ_1_	0.258	9.37	536.4
SG-CTSQ_2_	0.413	9.11	497.2
SG-CTSQ_3_	0.475	8.62	445.9
SG-CTSQ_4_	0.537	8.28	403.1

**Table 2:** Adsorption kinetic parameters for Th(IV) on SG-CTSQ.

Adsorption Kinetic Model	Parameters	SG-CTSQ_1_	SG-CTSQ_2_	SG-CTSQ_3_	SG-CTSQ_4_
Pseudo first-order	q_e_ (mg·g^−1^)	10.15	15.37	22.35	25.86
	K_1_ (s^−1^)	0.053	0.028	0.031	0.034
	R^2^	0.995	0.973	0.957	0.924
Pseudo second-order	q_e_ (mg·g^−1^)	8.16	15.29	21.04	23.91
	K_2_ (g·s·mg^−1^)	0.014	0.006	0.004	0.006
	R^2^	0.964	0.971	0.981	0.993
Elovich	α_E_ (mg·(g·s)^−1^)	3.996	2.416	1.347	1.073
	β_E_ (g·mg^−1^)	0.685	1.152	2.837	2.158
	R^2^	0.797	0.837	0.882	0.906

**Table 3:** Langmuir and Freundlich adsorption isotherm parameters for Th(IV) on SG-CTSQ.

Adsorption Isotherm Model	Parameters	SG-CTSQ_1_	SG-CTSQ_2_	SG-CTSQ_3_	SG-CTSQ_4_
Langmuir	q_max_ (mg·g^−1^)	31.62	37.97	49.13	57.28
	B (L·mg^−1^)	0.029	0.064	0.053	0.085
	R^2^	0.957	0.982	0.987	0.992
Freundlich	K_f_	7.154	8.701	11.278	9.056
	n_f_	0.691	0.833	1.069	1.235
	R^2^	0.989	0.983	0.971	0.976

**Table 4:** Adsorption thermodynamic parameters of Th(IV) on SG-CTSQ.

Species	T (K)	∆G (KJ·mol^−1^)	∆H (KJ·mol^−1^)	∆S (J·mol^−1^·K^−1^)	R^2^
SG-CTSQ_1_	293.15	−2.85	17.47	69.31	0.9888
	303.15	−3.54			
	313.15	−4.23			
	323.15	−4.93			
	333.15	−5.62			
SG-CTSQ_2_	293.15	−2.13	20.93	78.68	0.9945
	303.15	−2.92			
	313.15	−3.71			
	323.15	−4.49			
	333.15	−5.28			
SG-CTSQ_3_	293.15	−2.02	22.45	83.47	0.9931
	303.15	−2.86			
	313.15	−3.69			
	323.15	−4.53			
	333.15	−5.36			
SG-CTSQ_4_	293.15	−0.25	26.91	92.65	0.9946
	303.15	−1.18			
	313.15	−2.11			
	323.15	−3.03			
	333.15	−3.96			

**Table 5:** Adsorption mechanism experiment results.

Dosage (mg)	C_e_ (mg·L^−1^)	Q_e_ (mg·g^−1^)	K_d_ = Q_e_/C_e_	lgK_d_	[SiNR_4_^+^] (mmol)	lg[SiNR_4_^+^]
20	17.51	12.44	0.71	−0.149	0.0107	−1.971
40	10.43	23.91	2.29	0.359	0.0215	−1.668
60	3.93	26.78	6.81	0.833	0.0322	−1.492
80	2.71	21.62	7.98	0.902	0.0429	−1.368
100	1.64	18.36	11.19	1.049	0.0537	−1.269

**Table 6:** Th(IV) recovery rate and decontamination factor.

Species	Recovery Rate	Decontamination Factor	Uranium Removal Rate
SG-CTSQ_1_	75.20%	291.8	99.74%
SG-CTSQ_2_	87.75%	339.5	99.73%
SG-CTSQ_3_	94.30%	368.8	99.74%
SG-CTSQ_4_	98.30%	385.1	99.75%

## Data Availability

All data are contained within the article.

## Supplementary Materials

The following supporting information can be downloaded at: https://www.mdpi.com/article/10.3390/molecules29133031/s1, Figure S1: The relationship between the partition coefficient Kd of various ions on TEVA and the concentration of nitric acid, Figure S2: Standard curve used for analyzing Th(IV) through X-ray fluorescence spectroscopy. Figure S3: The appearance images of SG, SG-CTS, and SG-CTSQ. Figure S4: Nitrogen sorption isotherm of SG-CTSQ_1_, SG-CTSQ_2_, SG-CTSQ_3_, and SG-CTSQ_4_. Supplementary Material S1: Characterizing instrument type and parameter.
