# Supplementary material for: Preparation of Quaternary Ammonium Separation Material based on Coupling Agent Chloromethyl Trimethoxysilane (KH-150) and Its Adsorption and Separation Properties in Studies of Th(IV)
Source: Molecules. 2024 Jun 26;29(13):3031. doi: 10.3390/molecules29133031 (PMC11243607; doi:10.3390/molecules29133031)
Supplement: Supplementary file 1 [file molecules-29-03031-s001.zip › molecules-3053461-supplementary.pdf]

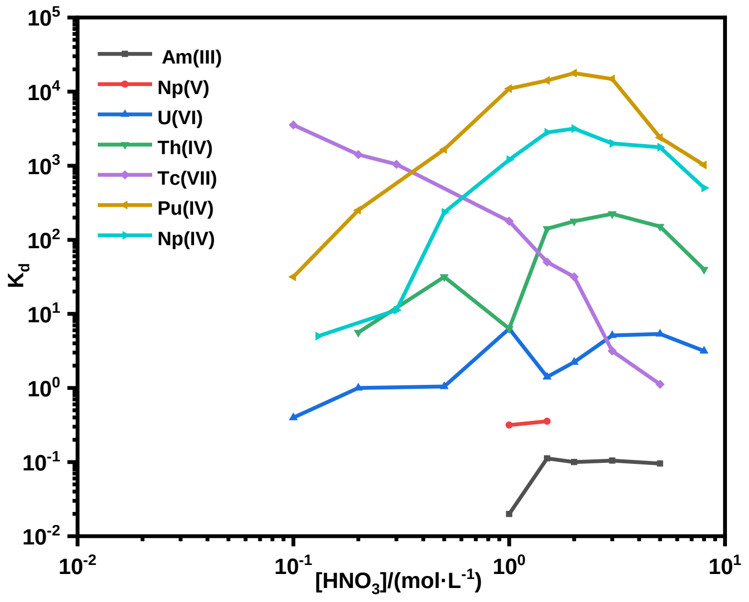

Figure S1. The relationship between the partition coefficient K_d of various ions on TEVA and the concentration of nitric acid.

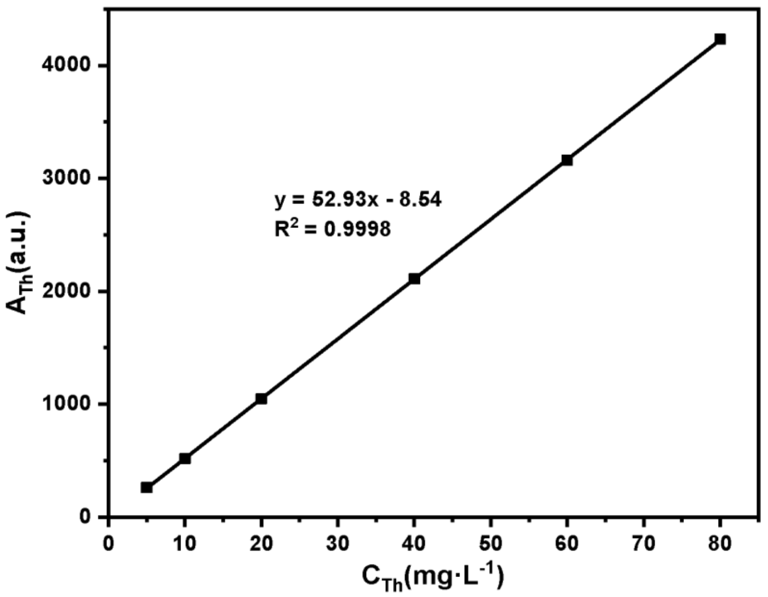

Figure S2. Standard curve used for analyzing Th(IV) through X-ray fluorescence spectroscopy.

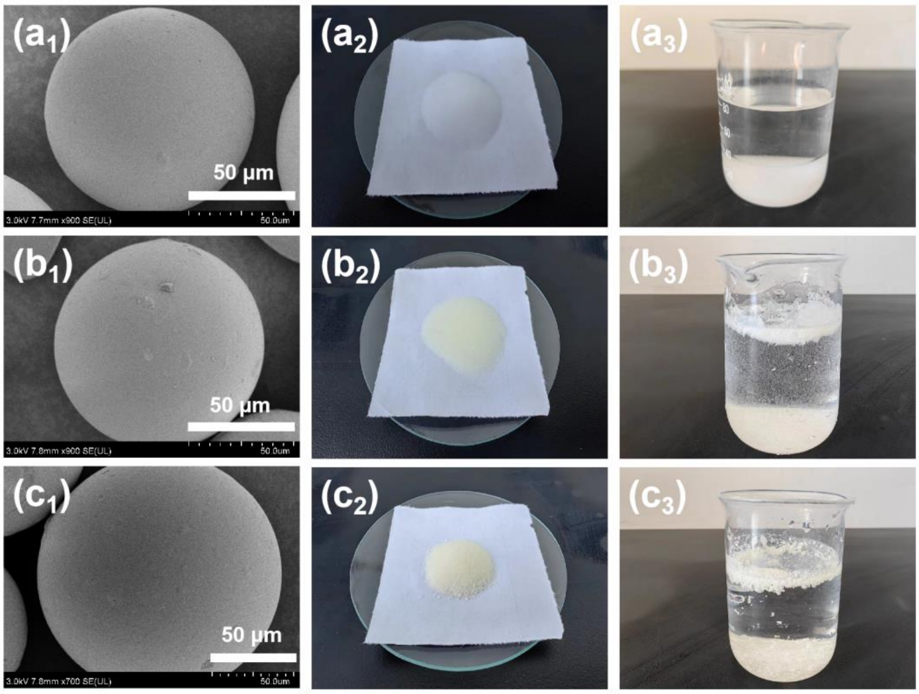

Figure S3. The appearance images of SG (a), SG-CTS (b), and SG-CTSQ (c).

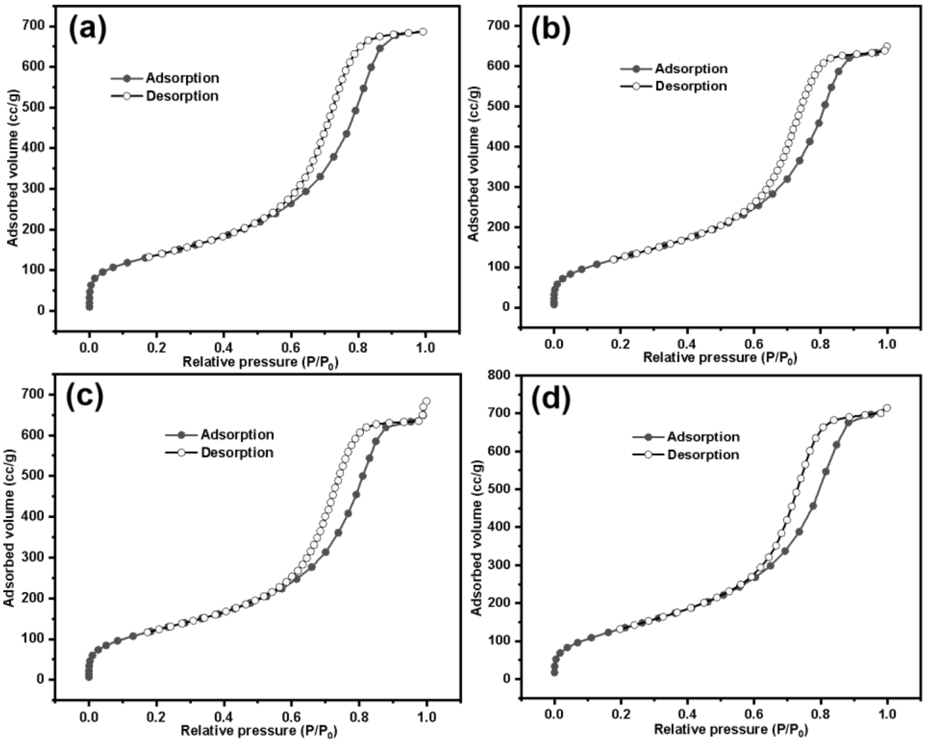

Figure S4. Nitrogen sorption isotherm of SG-CTSQ$_1$ (a), SG-CTSQ$_2$ (b), SG-CTSQ$_3$ (c), and SG-CTSQ$_4$ (d).

Supplementary Material S1:

Characterizing instrument type and parameter.

The S-4800 scanning electron microscopy (Hitachi, Tokyo, Japan) was used to image the microstructures of SG, SG-CTS and SG-CTSQ. Fourier transform infrared (FTIR) spectra were obtained on a IS50 FTIR spectrometer (Nicolet, Madison, WI, USA). The NMR spectra of SG and SG-CTS were measured on a Biospin Avance III spectrometer (Bruker, ^{29}Si, 600 MHz, USA). The NMR spectra of SG-CTS and SG-CTSQ were measured on a Biospin Avance III

spectrometer (Bruker, ^{13}C, 600 MHz, Boston, MA, USA). SG-CTS and SG-CTSQ underwent thermogravimetric analysis using the DSC3+ thermogravimetric analyzer (Mettler Toledo, Switzerland). Escalab 250Xi X-ray photoelectron spectrometer (XPS) (Thermo Scientific, Waltham, MA, USA) was used to measure surface element content and functional groups of SG, SG-CTS and SG-CTSQ. The specific surface area was determined using the Brunauer-Emmett-Teller (BET) method, while the pore diameter was calculated using the Barrett-Joyner-Halenda (BJH) method.
